# Treatment outcomes of MDR-tuberculosis patients in Brazil: a retrospective cohort analysis

**DOI:** 10.1186/s12879-017-2810-1

**Published:** 2017-11-14

**Authors:** Mayara Lisboa Bastos, Lorrayne Beliqui Cosme, Geisa Fregona, Thiago Nascimento do Prado, Adelmo Inácio Bertolde, Eliana Zandonade, Mauro N. Sanchez, Margareth Pretti Dalcolmo, Afrânio Kritski, Anete Trajman, Ethel Leonor Noia Maciel

**Affiliations:** 1 Social Medicine Institute, State University of Rio de Janeiro, Rio de Janeiro, RJ Brazil; 2 Public Health Post-Graduation Program, Federal University of Espírito Santo, Vitória, ES Brazil; 3 Statistical Department, Federal University of Espírito Santo, Vitória, ES Brazil; 4 Public Health Department, Brasília Federal University, Brasília, DF Brazil; 5 Oswaldo Cruz Foundation, Reference Center Hélio Fraga, Rio de Janeiro, Brazil; 6 Faculty of Medicine, Federal University of Rio de Janeiro, Rio de Janeiro, Brazil; 7 Brazilian Tuberculosis Network, Rio de Janeiro, Brazil; 8 McGill University, Montreal, Canada; 9 Rua Macedo Sobrinho 74/203, Humaitá 22271–080, Rio de Janeiro, Brazil

**Keywords:** Brazil, Tuberculosis, Multidrug-resistant, Treatment outcomes

## Abstract

**Background:**

Multidrug-resistant tuberculosis (MDR-TB) is a threat for the global TB epidemic control. Despite existing evidence that individualized treatment of MDR-TB is superior to standardized regimens, the latter are recommended in Brazil, mainly because drug-susceptibility tests (DST) are often restricted to first-line drugs in public laboratories. We compared treatment outcomes of MDR-TB patients using standardized versus individualized regimens in Brazil, a high TB-burden, low resistance setting.

**Methods:**

The 2007–2013 cohort of the national electronic database (SITE-TB), which records all special treatments including drug-resistance, was analysed. Patients classified as MDR-TB in SITE-TB were eligible. Treatment outcomes were classified as successful (cure/treatment completed) or unsuccessful (failure/relapse/death/loss to follow-up). The odds for successful treatment according to type of regimen were controlled for demographic and clinical variables.

**Results:**

Out of 4029 registered patients, we included 1972 recorded from 2010 to 2012, who had more complete outcome data. The overall success proportion was 60%. Success was more likely in non-HIV patients, sputum-negative at baseline, with unilateral disease and without prior DR-TB. Adjusted for these variables, those receiving standardized regimens had 2.7-fold odds of success compared to those receiving individualized treatments when failure/relapse were considered, and 1.4-fold odds of success when death was included as an unsuccessful outcome. When loss to follow-up was added, no difference between types of treatment was observed. Patients who used levofloxacin instead of ofloxacin had 1.5-fold odds of success.

**Conclusion:**

In this large cohort of MDR-TB patients with a low proportion of successful outcomes, standardized regimens had superior efficacy than individualized regimens, when adjusted for relevant variables. In addition to the limitations of any retrospective observational study, database quality hampered the analyses. Also, decision on the use of standard or individualized regimens was possibly not random, and may have introduced bias. Efforts were made to reduce classification bias and confounding. Until higher-quality evidence is produced, and DST becomes widely available in the country, our findings support the Brazilian recommendation for the use of standardized instead of individualized regimens for MDR-TB, preferably containing levofloxacin. Better quality surveillance data and DST availability across the country are necessary to improve MDR-TB control in Brazil.

**Electronic supplementary material:**

The online version of this article (10.1186/s12879-017-2810-1) contains supplementary material, which is available to authorized users.

## Background

Tuberculosis (TB) is still a leading cause of death globally. In 2015, 10 million new cases were reported worldwide [1]. Although highly effective treatments for TB are available, the control of the TB epidemics remains a challenge, mainly because of persisting global poverty, limited health access in many parts of the world, poor prevention programs and the emergence and rapid dissemination of the epidemics of HIV and of drug-resistant (DR)-TB [2].

Multidrug-resistant (MDR)-TB, defined as resistance to rifampicin (RIF) and isoniazid (INH), and extensively drug-resistant (XDR)-TB, defined as resistance to both drugs plus a second-line injectable drug and a fluoroquinolone, have become a threat in eastern Europe and some parts of Asia and sub-Saharan Africa. In 2015, 580,000 new MDR cases were reported, corresponding to 4% of all new cases, and 20% of previously treated TB cases [1]. With the implementation of new rapid diagnostic tests that detect at least RIF-resistance at baseline, more cases of DR-TB are likely to be detected. Treatment of DR-TB is longer, more expensive, more toxic and less effective, thus reducing quality of life [3].

While Brazil is one of the 30 high TB burden countries, the prevalence of DR-TB remains relatively low (7%), even after Xpert® MTB/RIF incorporation in the public health system [4]. Reasons for that might include TB treatment in Brazil being free of charge, standardized and only obtained in public health facilities, upon notification [2]. Quality-controlled drug supply and distribution are guaranteed by the Ministry of Health [2]. In addition, RIF has been used in fixed-dose combination (FDC) with INH since the 70s. Susceptible TB treatment is standardized (2RHZE/4RH) in FDC since 2010, and directly-observed treatment (DOT) is recommended for all TB cases [5]. Despite evidence from meta-analyses that individualized regimens are superior to standardized regimens for DR-TB treatment [6–9], recommended treatment for MDR in Brazil is also standardized, unlike most countries with a high burden of drug resistance [5].

One individual-patient [7] and four aggregate meta-analyses [6, 8–10] have studied the outcomes of different treatment regimens in MDR/XDR-TB. Most cohorts included in these meta-analyses were from high-burden MDR-TB countries, but the Brazilian cohort has never been included. Moreover, the relative role of different exposure variables, other than treatment, has been poorly explored in the literature, particularly in low MDR-TB prevalence settings. We aimed to analyse the impact of standardized treatment regimens adjusted for demographic and clinical variables in the treatment outcomes in a large cohort of Brazilian patients.

## Methods

### Patients’ selection and study design

This was an observational retrospective cohort study based on secondary data. Since 2010, the Brazilian National TB Program (NTP) has implemented an electronic database called “Information System on Special Treatments” (*Sistema de Informação de Tratamentos Especiais,* SITE-TB, http://sitetb.saude.gov.br/) [11] to record all Brazilian TB patients who receive “special” regimens, i.e., not the standard TB regimen (2RHZE/4RH), due to any of the following reasons: drug resistance, adverse events, non-tuberculosis mycobacterial disease or comorbidities incompatible with the TB standard regimen. In SITE-TB, demographic and clinical information (such as age, ethnic group, AIDS and diabetes), drug susceptibility test results (DST), adverse events, treatment regimens and outcomes for each patient are recorded and periodically updated according to clinical progress.

In August 2015, the SITE-TB database contained information from a cohort of anonymous patients diagnosed from January 2007 to December 2013 (some of whom started treatment in 2014 or 2015) with MDR-TB. To check the consistency of the information recorded in SITE-TB, we performed an exploratory analysis (Additional file 1: Table S1).

### Eligibility, inclusion and exclusion criteria

All patients classified as MDR-TB in the SITE-TB database were eligible to our study. Since the average duration of MDR-TB treatment is 2 years, many of the patients remained on treatment (38%) after January 2013, and only 17% of those had had a successful outcome (cure or completed treatment) reported. We thus excluded patients who started treatment after 2012, because of the risk of underestimating successful outcomes (classification bias). In addition, the number of patients reported in years 2007 to 2009 was notably inferior (Additional file 1: Table S1), mainly because the SITE-TB database was created in 2010, and the information from previous years was retrospectively inserted in the database. Thus, we also excluded patients reported before January 1, 2010 to avoid selection bias (e.g. patients with unsuccessful outcomes might not be reported or some regions of Brazil might not have reported cases). Therefore, only patients registered in the SITE-TB database between January 1, 2010 and December 31, 2012 were included.

The following additional exclusion criteria were adopted: (i) patients’ records with any inconsistent information: DST results for RIF missing or classified as susceptible, or patients initially classified as MDR-TB but with the final diagnosis changed to “not TB” and (ii) patients having received a standard treatment to susceptible TB (2RHZE/4RH) and having died before receiving treatment for MDR-TB.

### Treatment regimens for MDR-TB in Brazil

Brazilian guidelines recommend [5] treatment of MDR-TB with standardized regimens, mainly because DST in many settings of the Brazilian public free-of-charge health system is restricted to first-line drugs. The standardized regimens should include: (i) one fluoroquinolone (levofloxacin or ofloxacin), (ii) one injectable drug (streptomycin is the drug of choice; however, if resistance to streptomycin is confirmed or whenever it was used in a previous treatment, amikacin should be used), (iii) terizidone, and (iv) an oral first line drug (ethambutol or pyrazinamide), if susceptible. The duration of treatment ranges from 18 to 24 months, depending on clinical improvement and follow-up culture results. Individualized regimens are restricted to patients with additional resistance (additional first line drug resistance, pre-XDR-TB, XDR-TB), to patients who had adverse events with standardized regimens, or according to the physician’s experience. These regimens might include other oral drugs, such as clofazimine, linezolid, imipenem and high-dose isoniazid. We considered patients with an individualized treatment (according to DST) as a group since the regimen given to each patient is not specified in the database.

### Outcomes, exposure and statistical analysis

The Brazilian Guideline [5] classifies the end of MDR-TB treatment outcomes as:

(i) Cure: Three consecutive negative cultures (at months 12, 15 and 18), given a negative culture at the 12th month of treatment. If culture remains positive at the end of the 12th month, the next four consecutive cultures must be negative (at months 15, 18, 21 and 24).

(ii) Treatment completed: treatment completed without bacteriological evidence of either cure or failure, due to lack of bacteriological results.

(iii) On treatment: patient registered in the SITE-TB database as still receiving treatment in 2016.

(iv) Failure: if two or more of the three cultures after the 12th month are positive.

(v) Loss to follow-up: no patient attendance at the health facility for more than 30 consecutive days after the expected date of their return or, in the cases of supervised treatment, 30 consecutive days after the date of the last supervised dose.

(vi) Transfer out: transferred to another unit, and outcome is unknown.

(vii) Death due to TB: a patient whose death was caused by TB according to the death certificate and occurred during treatment.

(viii) Death due to other causes: a patient whose death was due to causes other than TB during TB treatment.

(ix) Changed regimen: Patients who changed to other treatment regimens due to adverse events, or due to a specific resistant pattern.

For the present study, we recoded outcomes according to Laserson’s recommendations [12]: all deaths were considered together as death due to any cause during the course of MDR-TB treatment; and “changed regimen” was considered a failure. In July 2016, we requested the Brazilian NTP to update the outcomes of patients recorded as “on treatment”. Since the average duration of MDR-TB treatment is two years and the included patients started treatment between 2010 and 2012, we recoded the patients who remained as “on treatment” as “loss to follow up”. The patients classified as “transfer out” had an unknown outcome related to their treatment, and were recoded as “loss to follow up” using the same approach of previous meta-analyses [7, 9, 13]. The original outcomes reported in the SITE-TB database are displayed in the descriptive results (Table 1 and Additional file 1 Table S1).

**Table 1:** Demographic and clinical variables of MDR-TB patients registered in SITE-TB, Brazil, 2007–2013

Variable	Included in study (1972)	Excluded from study (2057)	*P*- value
Sex			0.06
Female	662 (34%)	749 (36%)	
Male	1310 (66%)	1308 (64%)	
Age (mean)	39.5 (SD 13.5)	39.6 (SD 13.4)	0.89
Regimen			<0.01
Individualized	448 (23%)	562 (27%)	
Standardized	1524 (77%)	1466 (72%)	
Unknown	0 (0%)	29 (1%)	
Ethnical group			
Afro-Brazilian	1213 (61%)	1217 (59%)	0.04
Indigenous	10 (1%)	6 (1%)	
Caucasian	728 (37%)	795 (39%)	
Other	21 (1%)	39 (1%)	
TB Site			0.80
Pulmonary	1916 (97%)	1994 (97%)	
Extra-pulmonary	21 (1%)	21 (1%)	
Both	35 (2%)	42 (2%)	
AFB at baseline			
Negative	273 (14%)	293 (14%)	<0.01
Positive	1650 (84%)	1647 (80%)	
Unknown	49 (2%)	117 (6%)	
HIV			<0.01
Positive	180 (9%)	201 (10%)	
Negative	1721 (87%)	1697 (83%)	
Unknown	71 (4%)	159 (7%)	
Cavity			0.20
Yes	1554 (79%)	1665 (81%)	
No	396 (20%)	371 (18%)	
Unknown	22 (1%)	21 (1%)	
Bilateral disease			<0.01
Yes	1283 (65%)	1456 (70%)	
No	667 (34%)	580 (29%)	
Unknown	22 (1%)	21 (1%)	
Smoking			<0.01
Yes	156 (8%)	231 (11%)	
No/unknown	1816 (92%)	1826 (89%)	
Alcohol use			0.02
Yes	350 (18%)	424 (21%)	
No/unknown	1622 (82%)	1633 (79%)	
Diabetes			0.40
Yes	227 (12%)	218 (11%)	
No/unknown	1745 (88%)	1839 (89%)	
DOT			<0.01
Yes	1558 (79%)	1472 (72%)	
No/unknown	414 (21%)	585 (28%)	
Macro-region			0.08
North	223 (11%)	204 (9%)	
North-eastern	580 (29%)	560 (28%)	
Centre-west	51 (3%)	45 (2%)	
South-eastern	856 (44%)	981 (48%)	
South	262 (13%)	267 (13%)	
First DR-TB episode			<0.01
Yes	1643 (84%)	1510 (73%)	
No	284 (14%)	456 (23%)	
Unknown	45 (2%)	91 (4%)	
Outcome			<0.01
Cure*	686 (35%)	444 (22%)	
Complete treatment*	489 (25%)	430 (21%)	
Failure	183 (9%)	215 (10%)	
Loss to follow-up	389 (20%)	344 (17%)	
Death	211 (9%)	261 (13%)	
Unknown	7 (1%)	30 (1%)	
Transfer	0 (0%)	1 (0%)	
On treatment	7 (1%)	332 (16%)	
Six month culture conversion			<0.01
Yes	879 (45%)	795 (39%)	
No	206 (10%)	210 (10%)	
Unknown	887 (45%)	1052 (51%)	

Abbreviation: *DR-TB* drug-resistant tuberculosis, *AFB* acid fast bacilli, *SD* standard deviation, *DOT* directly observed treatment

^*^Among included patients 10 had relapse episodes of MDR-TB

Relapse was not recorded as an outcome in the original SITE-TB database. We searched for relapsed cases after treatment success among included patients, but we had no access to nominal data of patients diagnosed after 2013, therefore we could not search for relapsed cases in more recent years.

For the analyses, we further classified outcomes and compared success versus: i) failure or relapse (considered equivalent to an efficacy analysis); or ii) failure or relapse or death; iii) failure or relapse or death or loss to follow-up (considered equivalent to an effectiveness analysis).

Exposure variables were categorized; regimen was classified as standardized or individualized. The following clinical and demographic data were included in bivariate analyses: age, sex, ethnic group, smoking status, alcohol use, HIV infection, extension of disease (acid fast bacilli positivity at baseline, presence of cavities on chest radiography, and bilateral disease), previous history of DR-TB, DOT and diabetes. Variables associated with outcomes with ≤0.20 significance in the bivariate analyses entered the multivariate approach. We used a backward stepwise multivariate model. HIV co-infection and treatment regimen were maintained in the final model, regardless of significance. In the original SITE-TB database, alcohol abuse, smoking, diabetes and DOT are classified as “yes” versus “no/unknown” and we used these categories as available in our analysis.

The association of end of treatment outcomes and the use of individual drugs was examined only among patients who received standardized regimens. For this analysis, we performed a separate multivariate model, adjusting for variables found to be independently associated with outcomes in the main analyses, all of which are known to be associated with unfavorable outcomes: HIV infection, diabetes, extent of disease (sputum-smear at baseline, the presence of cavities on chest radiography and bilateral disease), and previous history of DR-TB.

The six-month sputum culture conversion – an intermediate outcome - was defined if the patient had two consecutive negative cultures at six months of treatment. Although we intended to use this variable as an outcome-dependent variable, it was used only as exposure, because the number of patients with performed cultures during the first six months of treatment was small.

Patterns of resistance other than MDR were poorly reported and were just described. They were not analyzed in any uni or multivariate model.

All statistical analyses were performed using SAS (version 9.4 Institute, Cary, NC, USA).

### Ethical approval

The study was reviewed and approved on 9 December 2014 (#906.298) by the institutional review board of *Universidade Federal do Espírito Santo*, which granted permission for use of the identified data for the purposes of the study and waived the need for written informed consent from participants as the study was based on secondary data and involved no more than minimal risk. All patients had an identification number, and to protect patients’ confidentiality, only one investigator (ELM) had access to both identified and de-identified codes; she prepared the anonymous database that was used in the study.

## Results

**Fig. 1:**
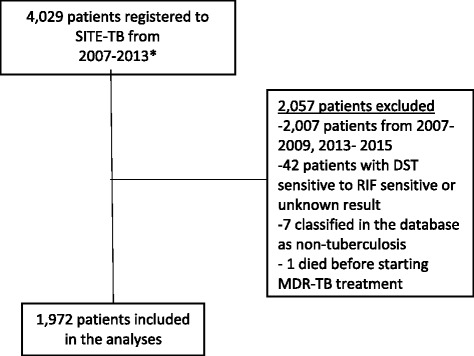
Flowchart of study population. Abbreviations: DST- Drug susceptibility test, MDR – Multi-drug resistant tuberculosis, SITE-TB- *Sistema de Informação de Tratamentos Especiais da Tuberculose* (National databank with all TB patients under regimens different from RHZE). *The dataset includes patients diagnosed from January 2007 to December 2013, some of whom started treatment in 2014 or 2015

As seen in Fig. 1, a total of 4029 MDR-TB patients were reported in SITE-TB, more than half of whom were excluded. Although there were statistically significant differences between the excluded and included populations in the study, the magnitude of differences in proportions was minor, and they concern mainly missing values, more frequent in the excluded group (Table 1)**.** The 1972 patients included had a mean age of 39.5 years (SD 13.5), 87% were male, 14% had previous DR-TB episodes, and 9% were HIV-infected (Table 1). Most patients had clinical features of advanced disease: 65% had bilateral disease, 84% were smear-positive at baseline, and 79% had cavities in chest radiographs (Table 1).

Regarding outcomes, 1175 patients (60%) had a successful outcome, with 389 loss to follow-up (20%). Among the 798 with unfavourable outcomes, 183 (9% of the total) had treatment failure, 211 (9%) died and 403 (22%) were loss to follow-up or had an unknown outcome or were still on treatment. Among patients who had a successful outcome, 10 relapsed subsequently with MDR-TB. Eight hundred and seventy-nine (45%) achieved culture conversion at six months (Table 1), of whom 534 (61%) were finally cured.

Most patients were treated with standardized regimens [*n* = 1524 (77%)]. The different standardized regimens are described in Additional file 1: Table S2. Most regiments had a total duration of 18 months, with an intensive phase in the initial 6 months; and all patients used regimens containing ethambutol and terizidone. Levofloxacin was more frequently used than ofloxacin (85% vs. 15%; respectively), and streptomycin was the injectable drug of choice in 51% of patients (Additional file 1: Table S2).

The resistance pattern of second-line and first-line drugs was poorly reported. Among the first-line drugs, besides RIF and INH, 29% of patients were resistant to streptomycin, 15% to pyrazinamide, and 25% to ethambutol (Additional file 1: Table S3).

Table 2 displays the bivariate analyses of factors associated with treatment outcomes, comparing success versus: i) failure/relapse or ii) failure/relapse/death; iii) failure/relapse/death/loss to follow-up. Compared to failure/relapse, success was more likely in patients who received standardized regimens, in non-smokers, with unilateral disease, in patients with a negative acid-fast bacillus (AFB) sputum smear at baseline, and in those who had a first episode of DR-TB. When “deaths” or “loss to follow-up” were added as unsuccessful outcomes, non-HIV patients had approximately a two-fold higher odds for treatment success, while non-diabetic patients were less likely to have a successful outcome. Regarding geographic macro-regions, the results considering the different outcomes were not consistent (Table 2).

**Table 2:** Factors associated with end of MDR-TB treatment outcomes in Brazil, univariate analysis

	Odds of treatment success (cure/completed) vs. failure/relapse *N* = 1358		Odds of treatment success (cure/completed) vs. failure/relapse/death *N* = 1569		Odds of treatment success (cure/completed) vs. failure/death/loss to follow-up N = 1972	
	Success (%)	OR (95% CI)	Success (%)	OR (95% CI)	Success (%)	OR (95% CI)
Regimen						
Standardized	922 (89%)	**2.8 (2.0; 3.8)**	922 (77%)	**1.7 (1.3; 2.2)**	922 (61%)	1.3 (1.0; 1.6)†
Individualized	243 (76%)	Reference	243 (66%)	Reference	243 (54%)	Reference
HIV co-infection*						
No	1053 (86%)	1.1 (0.6; 2.1)	1089 (75%)	**1.7 (1.2; 2.6)**	1089 (61%)	**2.2 (1.6; 3.0)**
Yes	76 (86%)	Reference	76 (64%)	Reference	76 (42%)	
Sex						
Female	402 (84%)	0.8 (0.6; 1.1)†	402 (74%)	0.9 (0.7; 1.2)	402 (61%)	1.1 (0.9; 1.4)
Male	763 (87%)	Reference	763 (75%)	Reference	763 (58%)	Reference
Age	–	1.0 (1.0; 1.1)	–	1.1 (1.0; 1.1)	–	1.1 (1.0; 1.1)
Macro-region						
North	153 (92%)	0.9 (0.4; 2.1)	153 (79%)	1.0 (0.6; 1.6)	153 (69%)	**1.6 (1.1; 2.3)**
Northeastern	309 (82%)	**0.3 (0.2; 0.6)**	309 (67%)	**0.5 (0.4; 0.8)**	309 (54%)	0.8 (0.6; 1.1)†
Centre-West	31 (86%)	0.6 (0.2; 2.0)	31 (82%)	1.2 (0.4; 2.8)	31 (61%)	1.1 (0.6; 2.0)†
Southeastern	519 (86%)	**0.5 (0.3; 0.9)**	519 (76%)	0.9 (0.6; 1.3)	519 (61%)	1.1 (0.8; 1.5)
South	153 (92%)	Reference	153 (80%)	Reference	153 (58%)	Reference
Smoking						
No/unknown	1087 (86%)	**1.7 (1.1; 2.9)**	1087 (75%)	1.5 (1.0; 2.2)†	1087 (60%)	**1.5 (1.1; 2.1)**
Yes	78 (79%)	Reference	78 (68%)	Reference	78 (50%)	Reference
Ethnical Group						
Afro-Brazilian	687 (86%)	1.0 (0.7; 1.3)	687 (73%)	0.9 (0.7; 1.1)	687 (57%)	**0.8 (0.6; 0.9)**
Indigenous	7 (88%)	1.1 (0.1; 8.9)	7 (88%)	2.1 (0.3; 17.6)	7 (70%)	1.4 (0.4; 5.5)
Other/unknown	15 (83%)	0.8 (0.2; 2.7)	15 (79%)	1.2 (0.4; 3.5)	15 (75%)	1.8 (0.6; 5.0)
Caucasian	455 (86%)	Reference	455 (76%)	Reference	455 (63%)	Reference
Alcohol use						
No/unknown	978 (86%)	1.1 (0.7; 1.6)	978 (75%)	1.2 (0.8; 1.6)	978 (60%)	1.3 (1.0; 1.6)†
Yes	187 (85%)	Reference	187 (72%)	Reference	187 (53%)	Reference
Diabetes						
No/unknown	1006 (85%)	0.7 (0.4; 1.2)†	1006 (74%)	0.8 (0.5; 1.1)†	1006 (57%)	**0.6 (0.4; 0.8)**
Yes	159 (90%)	Reference	159 (79%)	Reference	159 (70%)	Reference
Cavity‡						
No	241 (89%)	1.3 (0.8; 2.0)†	241(76%)	1.1 (0.9; 1.4)	241(61%)	1.1 (0.9; 1.4)
Yes	907 (85%)	Reference	907 (74%)	Reference	907 (58%)	Reference
Bilateral disease‡						
No	432 (88%)	**1.4 (1.1; 2.1)**	432 (80%)	**1.7 (1.3; 2.2)**	432(65%)	**1.5 (1.2; 1.8)**
Yes	716 (84%)	Reference	716 (71%)	Reference	716 (56%)	Reference
AFB positive§						
No	190 (93%)	**2.3 (1.3;4.1)**	190 (83%)	**1.7 (1.2; 2.5)**	190 (69%)	**1.7 (1.3; 2.2)**
Yes	960 (86%)	Reference	960 (74%)	Reference	960 (58%)	Reference
First DR-TB episode††						
Yes	1083 (91%)	**8.7 (5.9; 12.8)**	1083 (81%)	**5.6 (4.2; 7.8)**	1083 (66%)	**5.6 (4.2; 7.4)**
No	73 (52%)	Reference	73 (41%)	Reference	73 (26%)	Reference
**Number of previous treatment**	–	**0.2 (0.15; 0.26)**	–	**0.30 (0.20; 0.33)**	–	**0.26 (0.2; 0.33)**
DOT						
No/unknown	233 (83%)	0.6 (0.5; 1.0)†	233 (73%)	0.9 (0.7; 1.2)	233 (56%)	0.9 (0.7; 1.1)†
Yes	932 (87%)	Reference	932 (75%)	Reference	932 (59%)	Reference
Six month culture conversion **						
No	181 (95%)	1.0 (0.5; 2.1)	181 (93%)	1.0 (0.5; 1.9)	181 (87%)	1.3 (0.9;2.1)
Yes	741 (95%)	Reference	741 (93%)	Reference	741 (84%)	Reference
Quinolone used‡‡						
Levofloxacin	799 (90%)	1.5 (0.9; 2.5)†	799 (78%)	**1.6 (1.1; 2.3)**	799 (62%)	**1.5 (1.1; 1.9)**
Ofloxacin	123(86%)	Reference	123 (69%)	Reference	123 (53%)	Reference
Injectable used‡‡						
Streptomycin	503 (92%)	**1.7 (1.2; 2.6)**	503 (82%)	**1.8 (1.4; 2.4)**	503 (64%)	**1.4 (1.2; 1.7)**
Amikacin	419 (87%)	Reference	419 (77%)	Reference	419 (57%)	Reference
Pyrazinamide used‡‡						
No	147 (92%)	1.5 (0.8; 2.6)	147 (74%)	0.8 (0.6;1.1)†	147 (74%)	1.0 (0.8; 1.3)
Yes	775 (89%)	Reference	775 (78%)	Reference	775 (78%)	Reference

Abbreviations: *DR-TB* drug resistant tuberculosis, *AFB* acid fast bacilli

Footnotes:

Bold *p* value < 0.05

†p value <0.20

*Due to the missing values, the following number of patients were included: *N* = 1313 (cure vs. fail/relapse), *N* = 1517 (cure vs/fail/relapse/death), *N* = 1901 (failure/death/loss to follow-up)

‡Due to the missing values, the following number of patients were included: *N* = 1341 (cure vs. fail/relapse), *N* = 1552 (cure vs/fail/relapse/death), *N* = 1950 (failure/death/loss to follow-up)

§Due to the missing values, the following number of patients were included: N = 1329 (cure vs. fail/relapse), *N* = 1535 (cure vs/fail/relapse/death), *N* = 1923 (failure/death/loss to follow-up)

††Due to the missing values, the following number of patients were included: *N* = 1329 (cure vs. fail/relapse), *N* = 1527 (cure vs/fail/relapse/death), *N* = 1927 (failure/death/loss to follow-up)

**The following number of patients were included: *N* = 967 (cure vs. fail/relapse), *N* = 989 (cure vs/fail/relapse/death), *N* = 1085 (failure/death/loss to follow-up)

‡‡Among patients that used standardized regimens: *N* = 1033 (cure vs. fail/relapse), *N* = 1199 (cure vs/fail/relapse/death), N = 1524 (failure/death/loss to follow-up)

In the final efficacy model (Table 3), the group receiving standardized regimens had 2.7-fold higher odds of successful treatment outcomes compared to those receiving individualized treatments. The odds were 1.4 higher when death was added to unsuccessful outcomes. In the effectiveness analysis, there was no significant difference in the odds of success for both types of treatments. AFB-negative sputum and first episode of DR-TB were consistently associated with more treatment success in the three analyses. Absence of HIV infection, of bilateral disease and presence of diabetes were associated with more successful outcomes only if death (for HIV and bilateral disease) and loss to follow-up (for the three exposure variables) were also considered. Finally, patients from specific macro-regions of Brazil (Northeastern and Southeastern) had lower odds of successful treatment.

**Table 3:** Factors associated with end of MDR-TB treatment outcomes in Brazil, multivariate analysis

	Adjusted odds of treatment success (cure/completed) vs. failure/relapse (*N* = 1,302) OR (95% CI)	Adjusted odds of treatment success (cure/completed) vs. failure/relapse/death (*N* = 1,483) OR (95% CI)	Adjusted odds of treatment success (cure/completed) vs. failure/relapse/death/lost to follow-up (*N* = 1,864) OR (95% CI)
HIV			
No	1.7 (0.9; 3.4)	**2.7 (1.7; 4.2)**	**2.3 (1.6; 3.4)**
Yes	Reference	Reference	Reference
Regimen			
Standardized	**2.7 (1.8; 3.9)**	**1.4 (1.1; 1.9)**	1.0 (0.8; 1.3)
Individualized	Reference	Reference	Reference
First DR-TB episode			
Yes	**8.8 (5.7; 13.5)**	**5.4 (3.8; 7.7)**	**2.1 (1.1; 4.1)**
No	Reference	Reference	Reference
AFB positive			
No	**4.2 (2.1; 8.2)**	**2.1 (1.4; 3.1)**	**2.1 (1.5; 2.8)**
Yes	Reference	Reference	Reference
Bilateral disease	–		
No		**1.4 (1.1.; 1.9)**	**1.3 (1.1; 1.6)**
Yes		Reference	Reference
Number of previous regimens	–	–	**0.4 (0.2; 0.7)**
Diabetes	–	–	
No/Unknown			**0.7 (0.5; 0.9)**
Yes			Reference
Macro-region			–
North	0.9 (0.3; 2.2)	0.9 (0.5; 1.5)	–
Northeastern	**0.2 (0.1; 0.5)**	**0.4 (0.4; 0.7)**	
Centre-West	0.4 (0.1; 1.8)	1.0 (0.4; 2.6)	
Southeastern	**0.4 (0.2; 0.8)**	0.7 (0.5; 1.1)	
South	Reference	Reference	

The variables that had a *p*- value <0.2 in the bivariate analysis entered in the initial multivariate-model (see Table 2). We performed a separate multivariate model for each group of outcome. Only variables that remained in the final model are shown. Bold font indicates statistically significant results (*p*-value < 0.05)

*Abbreviations*: *DR-TB* Drug resistant tuberculosis, *AFB* acid fast bacilli

Regarding individual drugs within standardized regimens, levofloxacin was superior to ofloxacin when success was compared to failure/relapse/death or failure/relapse/death/loss to follow-up. Regarding the type of injectable drug used (i.e. amikacin vs. streptomycin) and the use of pyrazinamide, no significant differences were observed considering any combination of unfavorable treatment outcomes (Additional file 1: Table S4).

## Discussion

In this study of a Brazilian TB-MDR cohort with nearly 2000 patients, we found lower rates of successful treatment (60%) than those recommended by the World Health Organization (WHO) (75%), mainly due to a high proportion of follow-up losses (20%). Not surprisingly, treatment success was more likely in non-HIV patients, in those with a first episode of DR-TB, sputum-negative at baseline and with unilateral disease. Conversely, non-diabetic patients and patients from specific macro-regions of Brazil were less likely to have successful outcomes. Adjusted for these variables, patients who received one of the Brazilian standardized regimens had nearly 3-fold higher odds of success in the efficacy analysis (excluding deaths) and among them, those who used levofloxacin instead of ofloxacin had nearly two-fold higher odds of success, with marginal significance, in the effectiveness analysis, although not in the efficacy analysis.

Although comparable to previously reported success rates in MDR-TB patients [6–10], we may have overestimated the proportion of cure in our cohort [14]. We only included patients who started treatment between 2010 to 2012. Considering at least 18-months of treatment with a relatively short follow-up interval, and the absence of nominal data for a linkage-based search in recent years, relapsed cases may have been underestimated. The high losses to follow-up despite high DOT coverage may be due to the different definitions and types of DOT across the health facilities in the country. Previously published studies have shown heterogeneous effects of DOT on MDR-TB outcomes, depending on whether the DOT was throughout therapy and whether it was home- or facility-based [8, 15, 16].

The effect of the standardized regimens on treatment outcomes should be interpreted with caution. The benefit in efficacy observed in the present study contrasts with three previous aggregate meta-analyses, in which patients who received individualized regimens had higher rates of treatment success [6, 8, 9]. These meta-analyses’ authors discussed that aggregate data may not be the ideal source for decisions on the choice of MDR-TB regimens. In addition, the higher odds of successful treatment among patients who received standardized regimens observed in our study could be due to selection bias or confounding factors that we were unable to assess. The choice of individual regimen may have been due to more severe clinical status or resistance patterns. Patients who received individualized regimens could have experimented adverse events, or might have received a tailored regimen due to extra-resistance beyond RIF and INH, which might not have been reported. The resistance to first- and second-line drugs are poorly reported and adverse events are not registered in SITE-TB.

Unfortunately, analyses of isolated drugs were only possible among patients using standardized regimens. However, we still had a large cohort in this category. Patients who used levofloxacin were 1.7-fold more likely to cure when compared to ofloxacin. The use of later generation quinolones (i.e. moxifloxacin, high-dose levofloxacin, and gatifloxacin) have indeed been found to be superior, [7] and have been incorporated in regimens recommended by WHO [17] and by the Brazilian NTP [5] . Conversely, the benefit of pyrazinamide in DR-TB has been observed when the *M. tuberculosis* strain was proven to be susceptible to that drug [13]. Currently, WHO recommends the use of pyrazinamide as an add-on agent, and it is not considered one of the core second-line agents. In our study, the use of pyrazinamide had no impact in treatment outcomes. However, we could not adjust our analysis for resistant patterns, since the DST for pyrazinamide was not reported in 65%.

Few studies have reported the effect of clinical variables on MDR-TB treatment outcomes. Clinical variables might have a substantial impact in drug- susceptible-TB outcomes. Some of these clinical characteristics, such as indirect signs of advanced disease (bilateral lesions, presence of cavitation and positive sputum at baseline) and of severity of resistance (previous episodes of DR-TB) were consistently associated with unsuccessful outcomes, no matter which outcomes were included in the analyses. These findings highlight the importance of early diagnosis of MDR-TB. We could not evaluate time elapsed from diagnosis to MDR-TB treatment initiation, due to unreliable information of the date of MDR diagnosis.

Non-HIV patients had a nearly three-fold higher chance of successful treatment when death was added to the unsuccessful definition. Poor TB outcomes are expected in HIV-patients, with higher mortality rates [18–23]. Timing of anti-retroviral therapy (ART) in TB/HIV patients is controversial, because of the risk of the immune reconstitution syndrome, which can accelerate TB progression and even be fatal [24, 25]. Unfortunately, we had no information on ART and its timing in MDR-TB/HIV patients.

This study has a number of limitations. In addition to the inherent limitations of observational retrospective studies, the available data had a few other problems. Missing information about several variables or not ready-to use information about isolated drugs were important bottlenecks for our analyses, reducing the number of patients included in the multivariate model. However, because we had a large initial dataset, we still had power to find significant associations, the benefit of standardized regimens being the most relevant and original.

Another limitation was the reporting of variables such as diabetes, alcohol consumption and smoking as “yes” and “no”; in which missing data were reported as “no”, generating a possible misclassification bias. Moreover, no standard definitions of smoking and alcohol use were available, nor information regarding insulin-dependency in diabetic patients. These co-morbidities are known predictors of poor outcomes in MDR-TB patients [26–30]; thus, it is plausible that the lack of or the inverse association of these variables with successful treatment outcomes might be due to the limitations of our data set.

Finally, nearly half of the cases in our cohort did not have information about culture results during the first six-months of treatment. Thus, the analysis of the six-month culture conversion rate as an intermediate outcome was jeopardized. The identification of predictors of culture conversion is important because it could affect the duration of the intensive phase (i.e. the use of injectable drugs) and could be used as a predictor of cure [31, 32].

Despite these limitations, our inclusion criteria allowed the minimization of outcome biases. In addition, the adjustment of the analyses to relevant clinical variables reduced the risk of confounding, albeit with limitations. Using a large cohort of MDR-TB, we found that adjusting for relevant demographic and clinical variables, standardized regimens were superior to individualized regimens, preferably with levofloxacin.

## Conclusion

Since randomized trials in MDR-TB patients are scarce, until stronger evidence is produced and DST for first- and second-generation drugs become widely available in the country, our findings support the Brazilian recommendation for the use of standardized regimens for MDR-TB. Levofloxacin rather than ofloxacin should be the quinolone of choice. Better quality surveillance data across the country are needed to improve MDR-TB control in Brazil. Improvement of data reporting in the national database (SITE-TB) is essential, including ART initiation, culture results, more accurate data on diabetes, alcohol abuse, DOT definitions, smoke use and the use of individual drugs.

## Additional file

Additional file 1: Table S1. Descriptive analysis of treatment outcomes and the year of treatment started in all eligible patients (*N* = 4029). **Table S2.** Type of standardized regimens of included patients. **Table S3.** Resistance pattern of patients included in the study (*n* = 1972). **Table S4.** The association of use of individual drugs and treatment outcomes (DOCX 108 kb)

## Abbreviations

AFB: Acid fast bacilli
ART: Anti-retroviral therapy
DOT: Directly observed treatment
DR: Drug-resistant
DST: Drug susceptibility test results (DST)
FDC: Fixed-dose combination
INH: Isoniazid
MDR-TB: Multidrug-resistant tuberculosis
NTP: National TB Program
RIF: Rifampicin
SITE-TB: Sistema de Informação de Tratamentos Especiais
TB: Tuberculosis
WHO: World Health Organization
XDR-TB: Extensively- drug-resistant tuberculosis
