# Supplementary material for: Treatment outcomes of MDR-tuberculosis patients in Brazil: a retrospective cohort analysis
Source: BMC Infect Dis. 2017 Nov 14;17:718. doi: 10.1186/s12879-017-2810-1 (PMC5686842; doi:10.1186/s12879-017-2810-1)
Supplement: Additional file 1: Table S1. — Descriptive analysis of treatment outcomes and the year of treatment started in all eligible patients (N = 4029). Table S2. Type of standardized regimens of included patients. Table S3. Resistance pattern of patients included in the study (n = 1972). Table S4. The association of use of individual drugs and treatment outcomes (DOCX 108 kb) [file 12879_2017_2810_MOESM1_ESM.docx]

**Supplementary Tables for**

**“Treatment outcomes of MDR-tuberculosis patients in Brazil: a retrospective cohort analysis”**

**Table S1- Descriptive analysis of treatment outcomes and the year of treatment started in all eligible patients (N=4,029)**

| Year | Complete treatment | Cure | Death | Failure | Loss to follow-up | On treatment | Transfer | Unknown | Total |
| --- | --- | --- | --- | --- | --- | --- | --- | --- | --- |
| 2007 | 103 (27%) | 93 (25%) | 66 (18%) | 43 (12%) | 61 (17%) | 0 (0%) | 0 (0%) | 2 (1%) | 368 |
| 2008 | 123 (32%) | 100 (26%) | 67 (18%) | 34 (9%) | 48 (13%) | 0 (0%) | 0 (0%) | 6 (2%) | 378 |
| 2009 | 124 (31%) | 115 (28%) | 63 (15%) | 43 (10%) | 58 (14%) | 4 (1%) | 0 (0%) | 5 (1%) | 412 |
| 2010 | 173 (29%) | 194 (32%) | 66 (11%) | 57 (9%) | 112(19%) | 0 (0%) | 0 (0%) | 1 (0%) | 603 |
| 2011 | 184 (26%) | 241 (35%) | 77 (11%) | 64 (10%) | 122 (18%) | 1 (0%) | 0 (0%) | 2 (0%) | 691 |
| 2012 | 140 (19%) | 264 (36%) | 73 (10%) | 72 (10%) | 161 (22%) | 6 (1%) | 0 (0%) | 12 (2%) | 728 |
| 2013 | 72 (10%) | 123 (19%) | 52 (7%) | 79 (11%) | 153 (21%) | 224 (31%) | 1 (0%) | 9 (1%) | 712 |
| 2014 | 0 (0%) | 0 (0%) | 8 (6%) | 6 (5%) | 18 (14%) | 98 (75%) | 0 (0%) | 0 (0%) | 130 |
| 2015 | 0 (0%) | 0 (0%) | 0 (0%) | 0 (0%) | 0 (0%) | 6 (100%) | 0 (0%) | 0 (0%) | 6 |

**Table S2 – Type of standardized regimens of included patients**

| Standardized | N=1,524 |
| --- | --- |
| 2Am_5_CfzEOfxTrd - 10Am_2_CfzEOfxTrd / 6CfzEOfxTrd | 2 (0.5%) |
| 2S_5_CfzEOfxTrd -10S_2_CfzEOfxTrd / 6CfzEOfxTrd | 2 (0.5%) |
| 2Am_5_EOfxZTrd - 4Am_2_EOfxZTrd - 6Am_2_EOfxTrd / 6EOfxTrd | 4 (1%) |
| 2S_5_EOfxZTrd - 4S_2_EOfxZTrd - 6S_2_EOfxTrd / 6EOfxTrd | 1 (0%) |
| 2S_5_EOfxTrd - 4S_3_EOfxTrd / 12EOfxTrd | 16 (1%) |
| 2S_5_EOfxZTrd - 4S_3_EOfxZTrd / 12EOfxTrd | 60 (4%) |
| 2S_5_ELfxTrd - 4S_3_ELfxTrd / 12ELfxTrd | 83 (5%) |
| 2S_5_ELfxZTrd - 4S_3_ELfxZTrd / 12ELfxTrd | 622 (40%) |
| 2Am_5_ELfxZTrd - 4Am_3_ELfxZTrd / 12ELfxTrd | 22 (1%) |
| 2Am_5_EOfxTrd - 4Am_3_EOfxTrd / 12EOfxTrd | 40 (4%) |
| 2Am_5_EOfxZTrd - 4Am_3_EOfxZTrd /12EOfxTrd | 108 (7%) |
| 2Am_5_ELfxTrd - 4Am_3_ELfxTrd / 12ELfxTrd | 100 (6%) |
| 2Am_5_ELfxZTrd - 4Am_3_ELfxZTrd / 12ELfxTrd | 464 (30%) |

**Abbreviations:** Am: Amikacin, E: Ethambutol, Cfz: Clofazimine, L: Levofloxacin, Ofx: Ofloxacin, S: Streptomycin, Trd: Terizidone, Z: Pyrazinamid

**Table S3-Resistance pattern of patients included in the study (n=1,972)**

| Drug | Resistant | Sensitive | Unknown |
| --- | --- | --- | --- |
| Rifampicin | 1972 (100%) | 0 | 0 |
| Isoniazid | 1967 (99%) | 4 (1%) | 1 (0%) |
| Ethambutol | 498 (25%) | 1338 (68%) | 136 (7%) |
| Pyrazinamide | 299 (15%) | 400 (20%) | 1273 (65%) |
| Streptomycin | 577 (29%) | 1249 (64%) | 146 (7%) |
| Amikacin | 19 (1%) | 228 (12%) | 1725 (87%) |
| Capreomycin | 17 (1%) | 200 (10%) | 1755 (89%) |
| Kanamycin | 13 (1%) | 200 (10%) | 1759 (89%) |
| Ofloxacin | 54 (3%) | 186 (9%) | 1732 (88%) |
| Ciprofloxacin | 5 (0%) | 11 (1%) | 1956 (99%) |
| Moxifloxacin | 5 (0%) | 8 (1%) | 1959 (99%) |
| Levofloxacin | 1 (0%) | 10 (1%) | 1961 (99%) |
| Ethionamide | 26 (1%) | 122 (6%) | 1824 (93%) |

**Table S4- The association of use of individual drugs and treatment outcomes**

|  | ^*^Adjusted odds of treatment success (cure/completed) vs. failure/relapse  (N=991) | ^*^Adjusted odds of treatment success (cure/completed) vs. failure/relapse/death  (N=1,185) | ^*^Adjusted odds of treatment success (cure/completed) vs. failure/death/loss to follow up  (N=1,457) |
| --- | --- | --- | --- |
| Quinolone used |  |  |  |
| Levofloxacin | 1.3 (0.7; 2.4) | **1.5 (1.1; 2.2)** | **1.4 (1.1; 1.9)** |
| Ofloxacin | Reference | Reference | Reference |
| Injectable used |  |  |  |
| Streptomycin | 1.2 (0.8; 1.9) | 1.3 (1.0; 1.9) | 1.0 (0.9; 1.4) |
| Amikacin | Reference | Reference | Reference |
| Pyrazinamide used |  |  |  |
| No | 2.1 (1.0; 4.6) | 0.9 (0.6; 1.3) | 1.2 (0.9; 1.6) |
| Yes | Reference | Reference | Reference |

**Footnote:**

^*^Models adjusted for extent of disease (AFB/bilateral diseases and cavities on chest radiography), diabetes, past history of treatment for drug-resistant TB and HIV co-infection.
